# Early fluid loading in acute respiratory distress syndrome with septic shock deteriorates lung aeration without impairing arterial oxygenation: a lung ultrasound observational study

**DOI:** 10.1186/cc13859

**Published:** 2014-05-06

**Authors:** Fabiola Prior Caltabeloti, Antoine Monsel, Charlotte Arbelot, Hélène Brisson, Qin Lu, Wen-Jie Gu, Guang-Ju Zhou, José O C Auler, Jean-Jacques Rouby

**Affiliations:** 1 Department of Surgery, Division of Anesthesiology, Hospital das Clínicas da Faculdade de Medicina da Universidade de São Paulo, 255 Dr Éneas de Carvalho Aguiar Ave, 05403-000 São Paulo, Brazil; 2 Laboratory of Clinical and Experimental Research of the Multidisciplinary Intensive Care Unit, Department of Anesthesiology and Critical Care Medicine, La Pitié-Salpêtrière Hospital, Assistance-Publique-Hôpitaux-de-Paris, UPMC Univ Paris 06, 47-83, Boulevard de l’Hôpital, 75013 Paris, France; 3 Department of Emergency Medicine, Second Affiliated Hospital, Zhejiang University, School of Medicine, 80 Jiefang Road, 310009 Hangzhou, China

## Abstract

**Introduction:**

The study was designed to assess the impact of fluid loading on lung aeration, oxygenation and hemodynamics in patients with septic shock and acute respiratory distress syndrome (ARDS).

**Methods:**

During a 1-year period, a prospective observational study was performed in 32 patients with septic shock and ARDS. Cardiorespiratory parameters were measured using Swan Ganz (n = 29) or PiCCO catheters (n = 3). Lung aeration and regional pulmonary blood flows were measured using bedside transthoracic ultrasound. Measurements were performed before (T0), at the end of volume expansion (T1) and 40 minutes later (T2), consisting of 1-L of saline over 30 minutes during the first 48 h following onset of septic shock and ARDS.

**Results:**

Lung ultrasound score increased by 23% at T2, from 13 at baseline to 16 (*P* < 0.001). Cardiac index and cardiac filling pressures increased significantly at T1 (*P* < 0.001) and returned to control values at T2. The increase in lung ultrasound score was statistically correlated with fluid loading-induced increase in cardiac index and was not associated with increase in pulmonary shunt or regional pulmonary blood flow. At T1, PaO_2_/FiO_2_ significantly increased (*P* < 0.005) from 144 (123 to 198) to 165 (128 to 226) and returned to control values at T2, whereas lung ultrasound score continued to increase.

**Conclusions:**

Early fluid loading transitorily improves hemodynamics and oxygenation and worsens lung aeration. Aeration changes can be detected at the bedside by transthoracic lung ultrasound, which may serve as a safeguard against excessive fluid loading.

## Introduction

Optimization of cardiac preload by fluid loading is a key issue in the treatment of septic shock. However, fluid administration may contribute to pulmonary edema in critically ill patients with acute respiratory distress syndrome (ARDS) whose alveolar-capillary permeability is increased. A positive hemodynamic response to fluid defined by a 10 to 15% increase in cardiac index (CI) [1,2], could be associated with arterial oxygenation deterioration and loss of lung aeration.

Two mechanisms may deteriorate gas exchange: increase in pulmonary shunt resulting from the increase in cardiac output (CO) and decrease in ventilation perfusion ratio induced by pulmonary edema resulting from an increase in pulmonary capillary wedge pressure (PCWP) [3,4]. On the other hand, because hypoxic pulmonary vasoconstriction is markedly impaired by lung and systemic inflammation [3], increase in venous mixed oxygen saturation (SvO_2_) may improve arterial oxygenation if pulmonary blood flow does not increase in non aerated lung regions. Therefore, the effects of fluid loading on arterial oxygenation are difficult to predict.

PCWP, SvO_2_, CO and intrapulmonary shunt (QVa/Qt) can be assessed at the bedside invasively, using a fiber optic Swan-Ganz catheter. Interstitial-alveolar edema, lung aeration and their variations can be assessed non-invasively using bedside transthoracic lung ultrasound [5-7]. Changes in pulmonary blood flow supplying non aerated lung regions can be evaluated using bedside Doppler lung ultrasound [8-11].

There are clear recommendations for fluid loading at the initial phase of septic shock [12] and a general agreement for restricting fluid intake at the early phase of ARDS. A retrospective study has suggested that adequate initial fluid resuscitation during the first 2 days followed by conservative late fluid management is essential for improving final outcome of patients with septic shock and ARDS [13]. No study however, has assessed the immediate effects of fluid administration on gas exchange and lung aeration. The primary objective of this study performed in patients with septic shock and ARDS was to evaluate the effects of fluid loading on lung aeration assessed by lung ultrasound. Secondary objectives were to assess concomitant changes in arterial oxygenation and hemodynamics.

## Material and methods

### Patients

This prospective observational study was performed in the multidisciplinary ICU of La Pitié-Salpêtrière hospital. The protocol was approved by the local Ethical Committee (*Comité de Protection des Personnes*, Ile de France VI, Groupe Hospitalier Pitié-Salpêtrière, Paris, France, session nº18/2011) who considered the study an integral part of care provided to patients with ARDS and septic shock and waived the need for patients to give written informed consent. In our ICU, such patients are routinely monitored using a PiCCO or a fiber optic Swan-Ganz catheter [14-16] and transthoracic lung ultrasound is a common procedure to assess changes in lung aeration [6,7,17]. Thirty-two mechanically ventilated patients were included within 48 h following onset of septic shock and ARDS, defined according to referent recommendations [18,19]. Patients’ relatives were orally informed of the study, including the results.

Exclusion criteria were age under 18 years, past history of chronic lung disease, active bleeding, ARDS and septic shock lasting more than 48 h, body positioning in the prone position, left ventricular ejection fraction lower than 50%, PCWP ≥18 mmHg, cardiogenic shock as the possible etiology, and pregnancy.

### Protocol

In each patient, fluid loading was directed by the attending physician following the criteria recommended by the Survival Sepsis Campaign: [12] within 6 h following onset of septic shock, 20 mL.kg^−1^ of saline were rapidly administered before norepinephrine administration; during the next 48 h, fluid loading was continued to obtain a central venous pressure (CVP) ≥12 mmHg and reduce the level of vasopressor support. At the time of inclusion, none of the patients had CVP ≥12 mmHg, thereby justifying the fluid loading they received. Patients were included between the 24th and the 48th hour. Twenty-nine patients included were previously monitored with an arterial catheter and a fiber optic pulmonary arterial catheter (Swan-Ganz Standard Thermodilution Pulmonary Artery Catheter, Edwards Lifescience, Irvine, CA, USA) and three with the PiCCO system (PULSION medical systems AG, Munich, Germany).

Respiratory, hemodynamic, echocardiographic and lung ultrasound measurements were performed at baseline before loading (T0). Then 1,000 mL of saline were infused over 30 minutes and, respiratory, hemodynamic and lung ultrasound parameters were measured at the end of volume expansion (T1) and 40 minutes later (T2). Arterial and mixed venous blood gas, red blood cell count, hematocrit and blood lactate were also measured at T0, T1 and T2.

All patients were sedated using midazolam 0.15 mg.kg^−1^.h^−1^ and sulfentanyl 0.2 μg.kg^−1^.h^−1^ and paralyzed using atracurium 50 mg.h^−1^ whenever it was needed [20]. Patients were ventilated using a volume-control mode with a constant inspiratory flow and a tidal volume of 5 to 6 mL.kg^−1^, the fraction of inspired oxygen (FiO_2_) was maintained at 0.6 or above if the arterial oxygen saturation (SaO_2_) remained <95%, the respiratory rate was optimized according to previous studies and recommendations [21] and the inspiratory/expiratory ratio was fixed at 0.5 (0.33 to 0.50). Positive end-expiratory pressure (PEEP) was set according to lung morphology as previously described [22,23]. Plateau pressure, PEEP and auto PEEP were recorded throughout the study. Ventilation settings were kept constant throughout the study.

### Measurements

Arterial pressure was continuously measured and recorded from the arterial line. In patients equipped with a fiber optic Swan-Ganz catheter pulmonary arterial pressure and right atrial pressure were continuously recorded on MetaVision Clinical Information Management System for ICUs (ICU electronic medical records system, iMDsoft, Tel-Aviv, Israel) and PCWP and CVP was intermittently measured at end-expiration. CO was measured with simultaneous withdrawing of systemic and pulmonary arterial blood samples within 1 minute. Arterial oxygen tension (PaO_2_), arterial carbon dioxide tension (PaCO_2_) and pH, hemoglobin and methemoglobin concentrations and arterial and mixed venous oxygen saturations were measured using an ABL 825 hemoximeter (Radiometer Copenhagen, Denmark). Standard formulas were used to calculate the CI, the systemic vascular resistance index (SVRI), pulmonary vascular resistance index (PVRI), the QVa/Qt, oxygen delivery (DO_2_), and oxygen consumption (VO_2_). In patients equipped with a PiCCO catheter, QVa/Qt and left ventricular cardiac filling pressures could not be measured.

In patients lying in the supine position or in the semi-recumbent position who were hemodynamically stable, echocardiographic measurements were performed offline, by an experienced observer with level-3 certification, who was unaware of the clinical data and other hemodynamic measurements. All measurements were made at end-expiration over five consecutive cardiac cycles. Transthoracic echocardiography was performed using a Siemens Acuson CV70 (Siemens Healthcare, Erlangen, Germany) and images were stored digitally for later playback and analysis. Left ventricular ejection fraction was assessed using the Simpson modified biplane method. Transmitral flow was recorded by pulsed Doppler with the sample volume placed at the mitral valve tips. Mitral inflow velocity was analyzed for peak velocity of early (E) and late (A) filling and deceleration time (DT) of E was measured. In the presence of arrhythmia, the peak velocity of early mitral inflow (E) was measured over five cardiac cycles [24]. Velocities of mitral annulus were recorded by a tissue Doppler imaging program with a 5-mm sample volume placed at the lateral corner of the mitral annulus. The early diastolic (E/e’) velocity of mitral annular displacement was measured from the tissue Doppler imaging recording.

**Figure 1:**
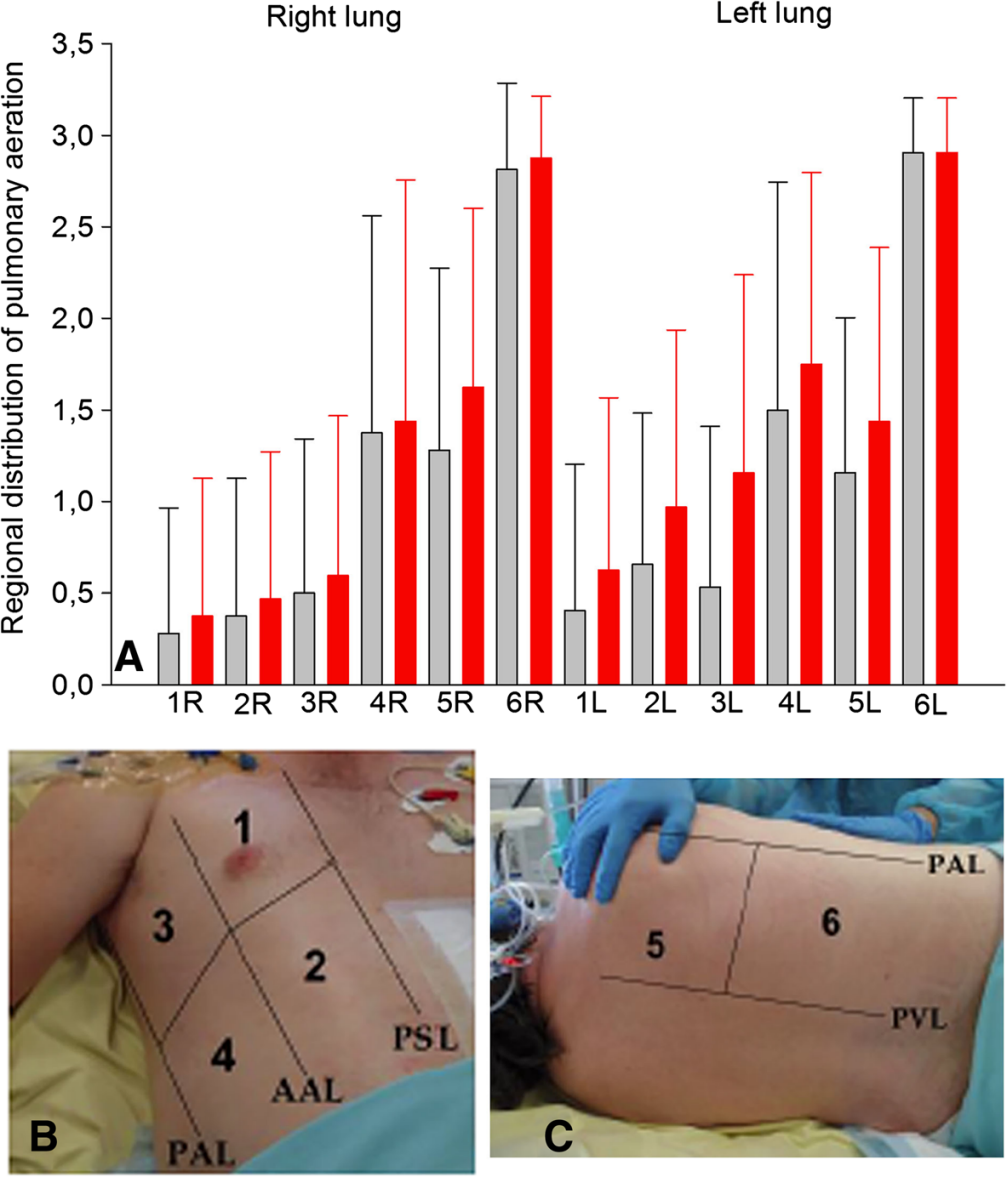
**Regional distribution of pulmonary aeration in 32 patients with septic shock and acute respiratory distress syndrome. (A)** Right (R, gray bars) and left (L, gray bars) lungs at baseline and 40 minutes after fluid loading (red bars). As shown in **(B)** and **(C)**, in each patient, six regions of interest were examined on each side, delineated by parasternal line (PSL), anterior axillary line (AAL), posterior axillary line (PAL) and paravertebral line (PVL): 1 = anterior and superior lung region; 2 = anterior and inferior lung region; 3 = lateral and superior lung; 4 = lateral and inferior lung region; 5 = posterior and superior lung region (the acoustic window is located in intercostal spaces between the scapula and vertebrae); 6 = posterior and inferior lung region. Each region of interest is characterized by the worst ultrasound pattern detected allowing the calculation of a regional ultrasound score: Normal = 0, interstitial edema (spaced B1 lines) = 1, interstitial-alveolar edema (coalescent B2 lines) = 2, lung consolidation = 3. The lung ultrasound score (LUS) is calculated as the sum of each individual score, ranging between 0 and 36 [27]. On the Y axis, the mean score per region of interest is indicated ± SD.

**Figure 2:**
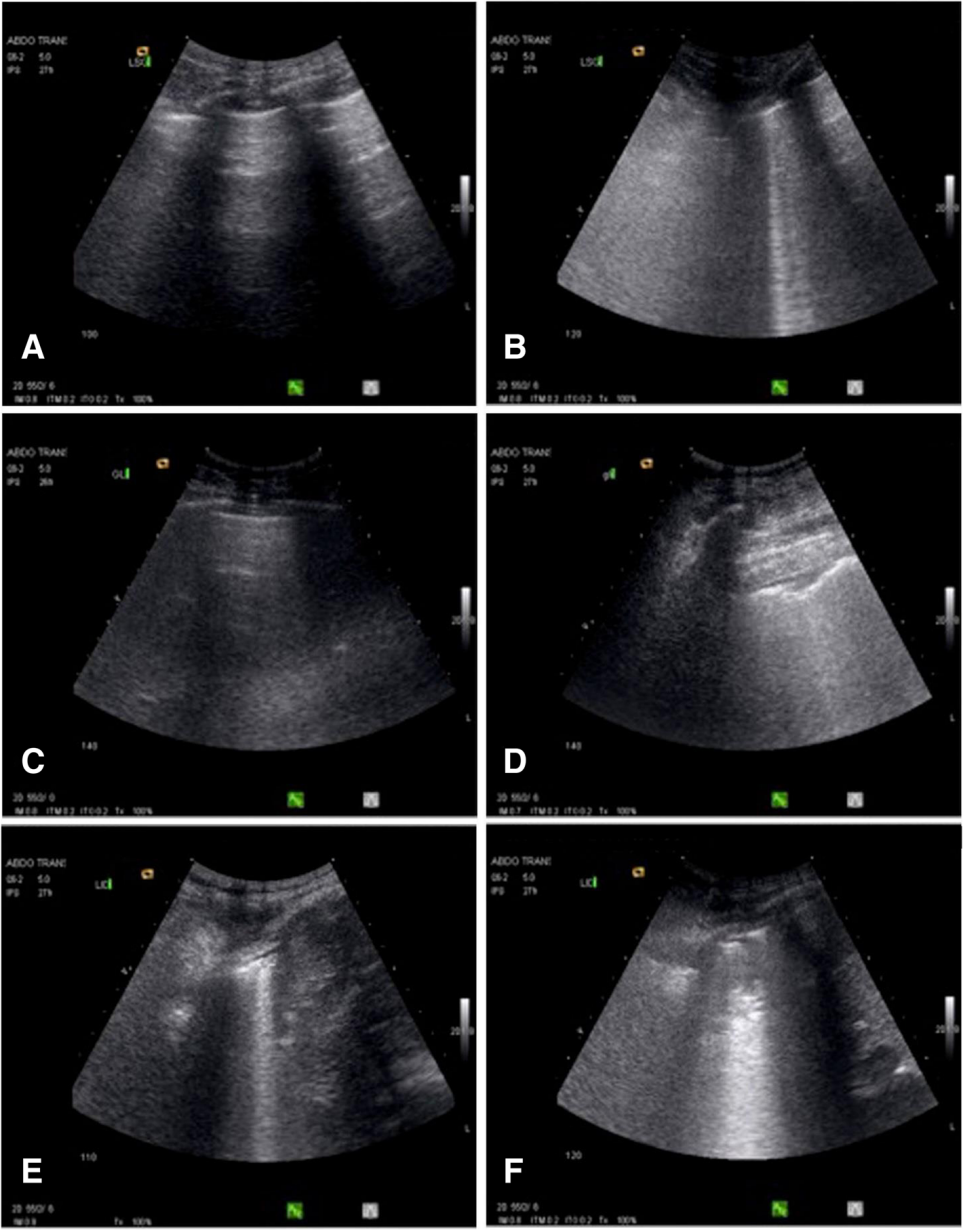
**Representative images illustrating lung ultrasound changes following fluid loading in three patients with septic shock and acute respiratory distress syndrome.** In the first patient, **(A)** the image obtained before fluid loading shows normal aeration in the left lateral and superior lung region with the presence of lung sliding and A lines; **(B)** the image obtained 40 minutes after fluid loading, shows the presence of B1 lines in the same lung region. In the second patient, **(C)** the image obtained before fluid loading shows normal aeration in the left lateral and inferior lung region with the presence of lung sliding and A lines; **(D)** the image obtained 40 minutes after fluid loading, shows the presence of B2 lines in the same lung region. In the third patient, **(E)** the image obtained before fluid loading shows B1 lines in the right lateral and inferior lung region; **(F)** the image obtained 40 minutes after fluid loading, shows the presence of B2 lines issued from a juxtapleural consolidation in the same lung region.

**Figure 3:**
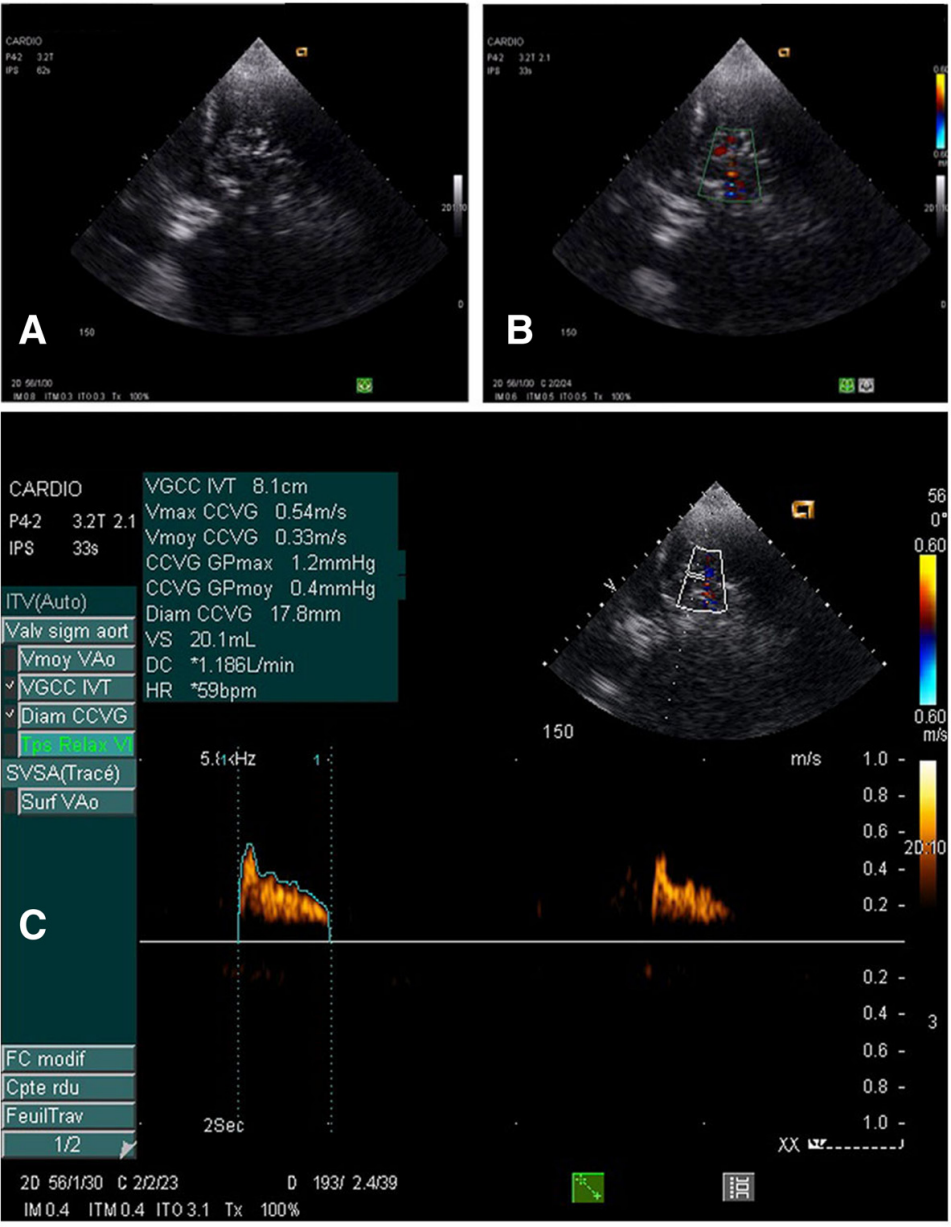
**Pulmonary blood flow. (A)** Lung consolidation with persisting regional pulmonary blood flow **(B)** detected using transthoracic Doppler ultrasound in a patient included in the study. Within the consolidation, a pulmonary artery characterized by its biphasic signal is identified **(C)**.

Transthoracic lung ultrasound was performed offline by an experienced investigator with level-3 certification [25], who was unaware of any other hemodynamic measures. A Siemens Acuson CV70 and a 2- to 4-MHz round-tipped or convex probe were used. As shown in Figure 1B and 1C, 12 regions of interest were examined and the quantification of aeration was calculated applying a lung ultrasound score (LUS) as previously described [26,27]. For a given region of interest, all intercostal spaces were carefully and extensively examined. Four ultrasound patterns corresponding to different degrees of aeration loss were looked for in each intercostal space: 0) normal aeration, characterized by the presence of lung sliding with horizontal *A lines* and, occasionally, one or two isolated vertical *B lines*; 1) moderate loss of lung aeration: either multiple well-defined and spaced B1 lines, issued from the pleural line or from small juxtapleural consolidations and corresponding to interstitial edema; or coalescent B2 lines, issued from the pleural line or from small juxtapleural consolidations, present in a limited portion of the intercostal space and corresponding to localized alveolar edema; 2) severe loss of lung aeration, characterized by multiple coalescent vertical B2 lines issued either from the pleural line (Figure 2D) or from juxtapleural consolidations (Figure 2F), detected in the whole area of one or several intercostal spaces and corresponding to diffuse alveolar edema; 3) complete loss of lung aeration resulting in lung consolidation and characterized by the presence of tissue pattern containing either hyperechoïc punctiform images representative of static air bronchograms, or hyperechoïc tubular images, representative of dynamic air bronchograms. The worst ultrasound pattern observed in one or several intercostal spaces was considered as characterizing the region of interest. A value (0, 1, 2 or 3) was attributed to each region examined and the LUS was calculated as the sum of the 12 regions examined. More detailed information on transthoracic lung ultrasound can be obtained online [28] by clicking on *Basic skills in transthoracic lung ultrasound* to download the corresponding PowerPoint presentation. Using Doppler with color-flow mapping, regional pulmonary blood flow present within consolidated lung areas was assessed as previously described [29]. Pulmonary arterial and venous blood flow signals were obtained by positioning the sample volume into the center of the lumen of the detected vessel and placing the ultrasound beam as parallel as possible to the flow. The spectral waveforms were considered good if 1) pulmonary artery blood flow could be differentiated from pulmonary vein blood flow; 2) the resolution was adequate for serial measurements. As previously described [9] arterial waveform consists principally of a rapid systolic acceleration phase, followed by rapid deceleration. Peak velocity, mean velocity and velocity time-interval were measured before and after fluid loading in the pulmonary artery supplying a consolidated lung region and characterized by a biphasic flow (Figure 3).

### Statistical analysis

The primary endpoint of the study was the increase in LUS after fluid loading. In a previous study, patients with ARDS had a mean LUS of 15 ± 4.8 [7]. Expecting a 30% increase in LUS resulting from fluid loading, we calculated that 31 patients would be needed based on 90% statistical power and an actual alpha value of 0.05. The secondary endpoints were changes in regional pulmonary blood flow, heart rate, mean arterial pressure, mean pulmonary arterial pressure, PCWP, right atrial pressure, CI, SvO_2,_ systemic and pulmonary vascular resistance indices, arteriovenous oxygen difference, oxygen delivery, oxygen consumption, lactate, hemoglobin, PaO_2_/FiO_2_ and QVa/Qt. Data are expressed as mean ± SD or median and interquartile range (25 to 75%) according to the data distribution. Cardiorespiratory variables, LUS, peak velocity, mean velocity and velocity time-intervals measured at T0, T1 and T2 were compared using one-way analysis of variance (ANOVA) for repeated measures or Friedman repeated measures ANOVA on ranks followed by post hoc Tukey test, using software Sigmastat 3.1 e Sigmaplot 11.0 (Systat Software Inc., Point Richmond, CA, USA). The statistical significance level was fixed at 0.05.

## Results

Clinical and cardiopulmonary characteristics of the patients are summarized in Table 1. According to the Berlin definition [19] 8 patients had mild ARDS, 21 moderate ARDS and 3 severe ARDS. Causes of ARDS were community-acquired pneumonia (n = 1), ventilator-associated pneumonia (n = 22), aspiration pneumonia (n = 1), pulmonary contusion (n = 2) and extrapulmonary ARDS (n = 6). Causes of septic shock were pneumonia (n = 26), peritonitis (n = 5) and necrotizing fasciitis (n = 1).

**Table 1:** Clinical and cardiorespiratory characteristics at baseline of the 32 patients with acute respiratory distress syndrome and septic shock

**Clinical characteristics**	
Age, yr	64 ± 13
Sex ratio, male/female, number	27/5
SOFA score	10 ± 2
APACHE II score	17 ± 6
**Causes of Admission**	
Emergency surgery	9
Elective surgery	15
Medical	4
Multiple trauma	4
**Hemodynamic characteristics**	
Swan-Ganz/PICCO, number of patients	29/3
Norepinephrine, number of patients	32
Norepinephrine dose, μg.Kg^1^.min^−1^, median (IQR)	0.34 (0.2 to 0.63)
Epinephrine, number of patients	1
LVEF%	60 (59 to 70)
**Respiratory Characteristics**	
LISS	2.3 ± 0.5
Vt, mL	400 (378 to 450)
Pmax, cmH_2_O	31 ± 7
Ppl, cmH_2_O	21 (17 to 24)
RR, breath.min^−1^	26 (22 to 29)
PEEP, cmH_2_O	8 (5 to10)
PEEP_i_, cmH_2_O	1 (0 to 2)
PaO_2_/FiO_2_	160 ± 49
pH	7.34 ± 0.08
PaCO_2_, mmHg	38.4 (34.4 to 42.1)

Data are presented as number of patients, mean ± SD, or median and interquartile interval (25 to 75%). SOFA, sequential organ failure assessment; APACHE II, acute physiology and chronic health evaluation; PICCO, PULSION medical systems; LVEF, left ventricular ejection fraction; E/A, ratio between early (E) and late (A) ventricular filling velocity; LISS, lung injury severity score; Vt, tidal volume; Pmax, peak pressure; Ppl, plateau pressure; RR, respiratory rate; PEEP, positive end-expiratory pressure; PEEPi, intrinsic PEEP; PaO_2_, arterial oxygen tension; FiO_2_, fraction of inspired oxygen; PaCO_2_, arterial carbon dioxide tension.

**Figure 4:**
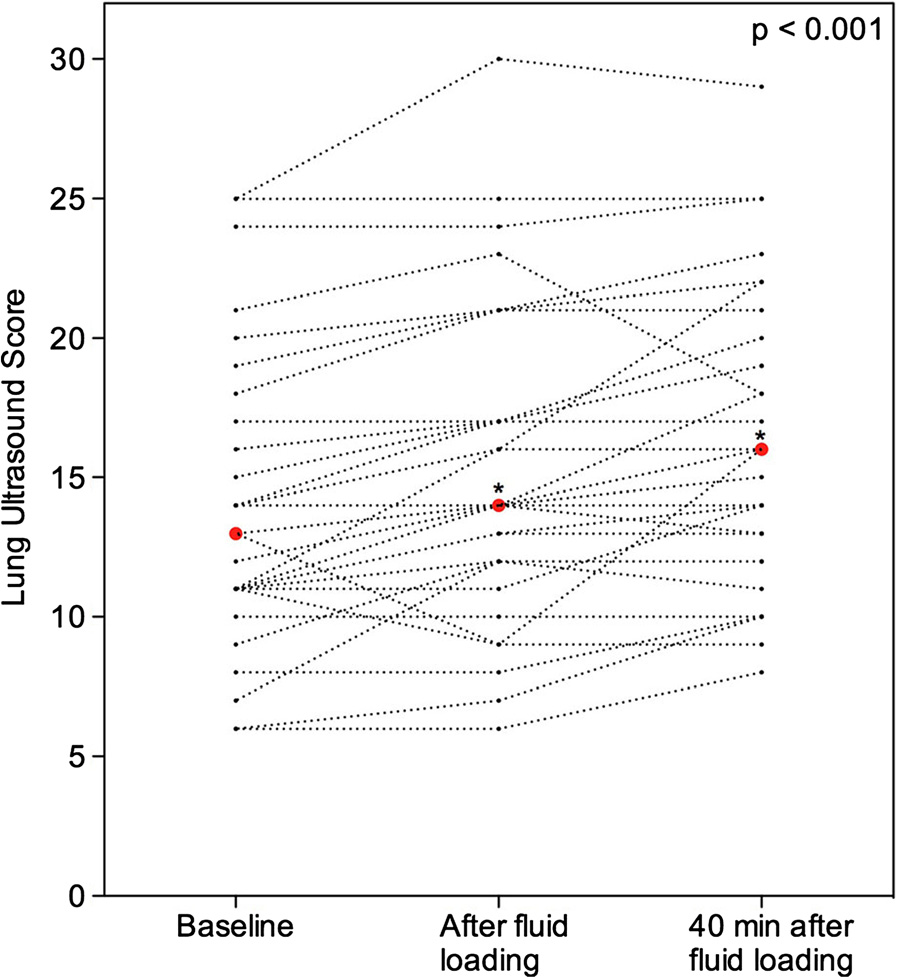
**Effects of fluid loading on lung ultrasound score in 32 patients with septic shock and acute respiratory distress syndrome.** Individual changes in lung ultrasound score are represented. Red dots represent median values. *P* <0.001 at the top of the figure indicates a statistically significant difference between the three time points using Friedman repeated measures analysis of variance on the ranks. Comparisons between two time points were performed using the post hoc Tukey test. *P <0.05, baseline versus end of 1,000-mL fluid loading and baseline versus 40 minutes after the end of fluid loading.

As previously reported in patients with ARDS using computerized tomography [30,31], loss of lung aeration was predominantly found in dependent posterior lung regions and similarly distributed on both sides (Figure 1A). Fluid loading-induced aeration loss observed between T0 and T2 was mainly due to the onset of coalescent B lines in initially normally aerated lung regions or regions with interstitial edema characterized by B1 lines (Figure 1A). As shown in Figure 4, LUS significantly increased by 8% between T0 and T1 (*P* = 0.01), and by 23% between T0 and T2 (*P* <0.001). New consolidations appeared only in 13% of lung regions concerned by aeration loss. Illustrative images of these changes are shown in Figure 2.

Table 2 summarizes hemodynamic changes observed between T0 and T2. CI, aortic velocity time-integral, mean arterial pressure, mean pulmonary artery pressure, PCWP, CVP, DO_2_ and VO_2_ increased significantly at T1 and returned to control values at T2 suggesting a redistribution of the fluid out of the vascular compartment. Concomitantly, systemic and pulmonary vascular resistance indices, arteriovenous oxygen difference, hemoglobin and lactates significantly decreased at T1 and returned to control values at T2.

**Table 2:** Hemodynamic changes before and after fluid challenge

**Hemodynamic variable**	**T0**	**T1**	**T2**	**p value**
HR, beats.min^−1^	96 (72,5 to 105.5)	93 (71.5 to 103.5)	91.5 (72.5 to 102.5)	0.067
PAM, mmHg	76.5 (71 to 83,6)	85 (76 to 90.5)^a,b^	81 (72.5 to 92)	<0.001*
PAPm, mmHg	25 (21 to 27)	28 (26 to 30)^a,b^	26 (24 to 29)^c^	<0.001*
PCWP, mmHg	12 (9.5 to 14)	14 (12 to 17)^a,b^	14 (10 to 14.2)	<0.001*
CVP, mmHg	10 (7.7 to 11.2)	12 (9.7 to 13)^a,b^	10 (8 to 11)	<0.001*
CI, l.min^−2^.m^−2^	3.4 ± 0.9	3.9 ± 0.9^a,b^	3.5 ± 1.0	<0.001*
SVRI, dyn.sec.cm^−5^ m^2^	1,576 (1,390 to 1,966)	1,427 (1,229 to 1,867)^a^	1,555 (1,290 to 2,229)	0.013*
PVRI, dyn.sec.cm^−5^ m^2^	311 (241 to 375)	262 (225 to 319)^a^	302 (265 to 403)^c^	<0.001*
SvO_2_,%	71 (67.8 to 78.6)	73.1 (68.1 to 78.8)	71.9 (68.1 to 78.4)	0.356
D (A-v)O_2_, mL/dL	3.5 (3.0 to 4.0)	3.3 (2.7 to 3.8)^a^	3.3 (2.9 to 3.9)	0.043*
DO_2_, mL.m^−1^.m^−2^	450 ± 127	489 ± 125^a,b^	456 ± 137	<0.001*
VO_2_, mL.m^−1^.m^−2^	118 ± 28	126 ± 29^a^	116 ± 31	0.03*
Lactate, mmol.L	1.9 (1.2 to 2.4)	1.55 (1.05 to 2.45)^a,b^	1.95 (1.15 to 2.27)	0.002*
Hb, g/dL	10 (8.8 to 10,9)	8.9 (8.1 to 10.5)^a^	9.6 (8.6 to 10.9)^c^	<0.001*
VTIAo, cm	18.3 ± 3.62	21.21 ± 4.49^a,b^	20.4 ± 4.63	<0.001*
E/A	1.05 ± 0.27	1.1 ± 0.31	1.11 ± 0.3	0.232
E/e’	5.17 (4.36 to 6.42)	6.27 (4.24 to 6.7)	5.65 (4.82 to 7.35)	0.657
Qva/Qt	25.3 (22.5 to 33)	26 (22.2 to 32.6)	27.5 (22.5 to 32.6)	0.307

Data are presented as mean ± SD or median and interquartile interval (25 to 75%). T0, baseline; T1, immediately after fluid challenge; T2, 40 minutes after fluid challenge; HR, heart rate; PAM, mean arterial pressure; PAPm, mean pulmonary artery pressure; PCWP, pulmonary capillary wedge pressure; RAP, right atrial pressure; CI, cardiac index; SvO_2_, mixed venous saturation; SVRI, systemic vascular resistance index; PVRI, pulmonary vascular resistance index; D(a-v)O_2_, arteriovenous oxygen difference; DO_2_, oxygen delivery; VO_2_, oxygen consumption; Hb, hemoglobin; VTIAo, aortic velocity time-integral; E/A, ratio of early (E) to and late (atrial - a) ventricular filling velocity; E/e’, ratio of early transmitral flow velocity to early diastolic mitral annulus velocity; Qva/Qt, pulmonary shunt. ^a^*P* <0.05 = T0 versus T1; ^b^*P* <0.05 = T1 versus T2; ^c^*P* <0.05 = T0 versus T2. *Statistically significant difference between the three time points.

**Figure 5:**
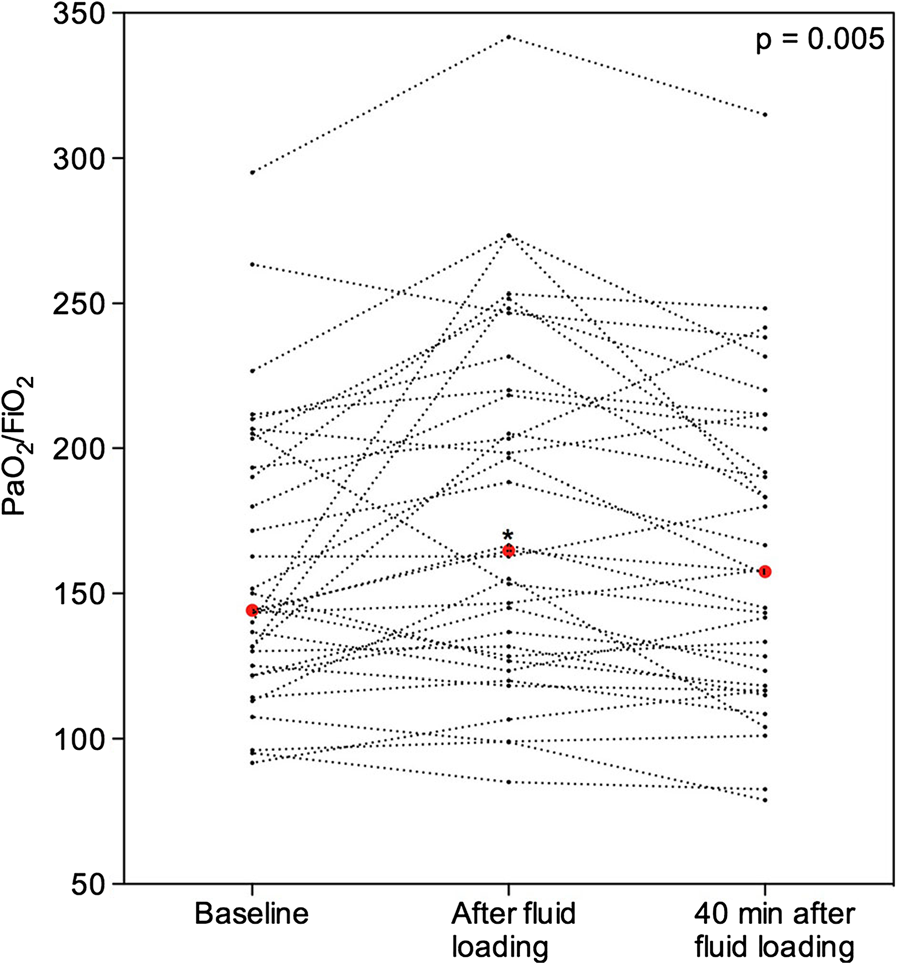
**Effects of fluid loading on arterial oxygenation in 32 patients with septic shock and acute respiratory distress syndrome.** Individual changes in arterial oxygen tension/fraction of inspired oxygen (PaO_2_/FiO_2_) are represented. Red dots represent median values. *P* = 0.005 at the top of the figure indicates a statistically significant difference between the three time points using Friedman repeated measures analysis of variance on the ranks. Comparisons between two time points were performed using the post hoc Tukey test. *P <0.05, baseline versus end of 1,000 mL fluid loading.

**Figure 6:**
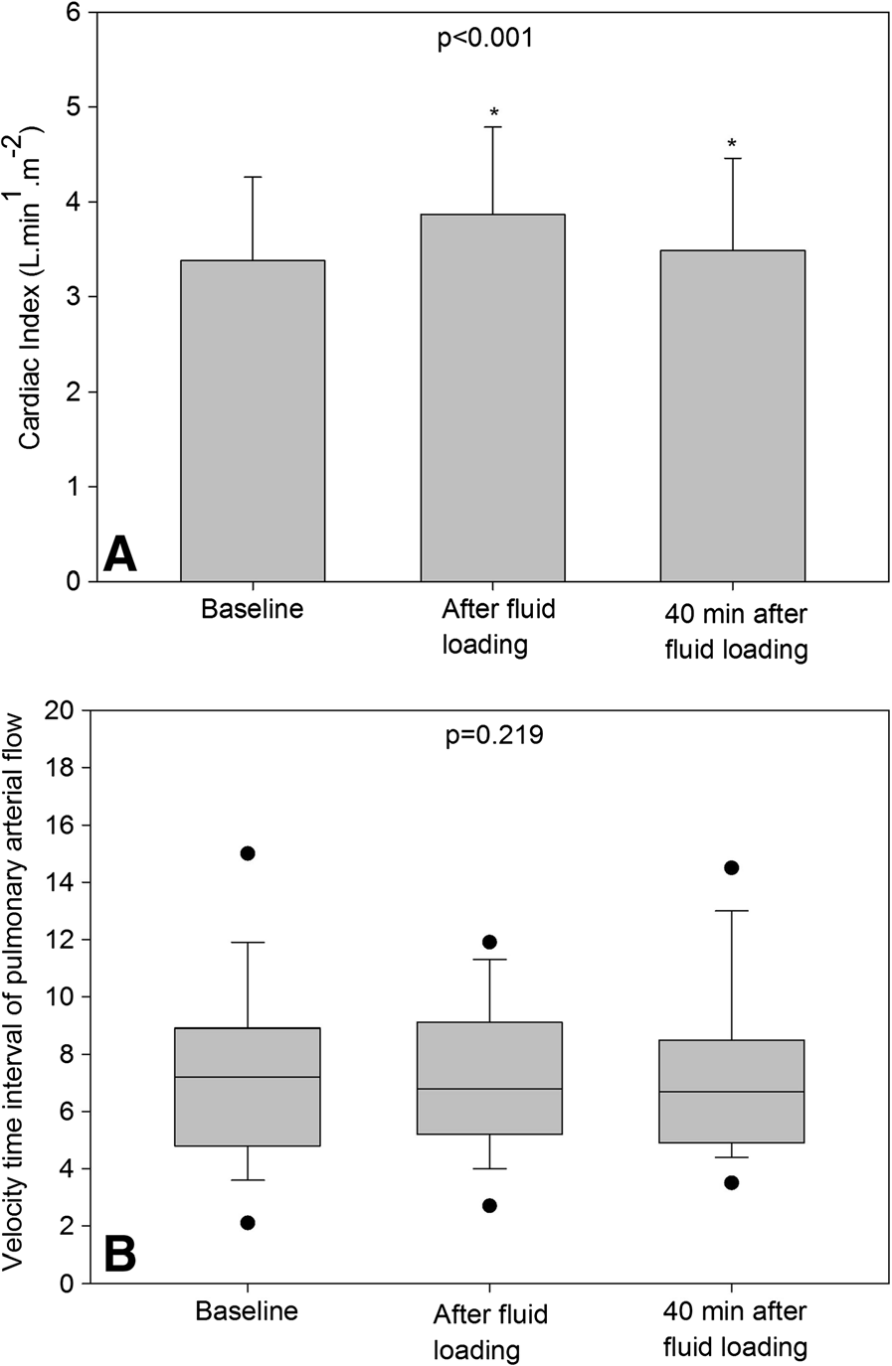
**Effects of fluid loading on cardiac index and pulmonary arterial blood flow supplying consolidated lung regions in 32 patients with septic shock and acute respiratory distress syndrome. (A)** Changes in cardiac index and **(B)** velocity time-interval of pulmonary arterial flow supplying consolidated lung regions following a 1,000-mL fluid loading are represented (mean ± SD or median and interquartile range, 25 to 75% according to distribution). *P* <0.001 at the top of the figure indicates a statistically significant difference between the three time points using analysis of variance for repeated measures. Comparisons between two time points were performed using the post hoc Tukey test. *P <0.05, baseline versus end of a 1,000-mL fluid loading and the end of 1,000-mL fluid loading versus 40 minutes after the end of fluid loading.

As shown in Figure 5, the PaO_2_/FiO_2_ ratio significantly increased at T1 and returned to control values at T2, whereas QVa/Qt remained unchanged (Table 2). Concomitantly, CI significantly increased at T1 and returned to control values at T2 (Figure 6A), whereas right and left velocity time intervals measured in the pulmonary arteries supplying non aerated lung regions, remained unchanged (Figure 6B). There was significant but weak correlation between the increase in LUS at T1 and the increase in CI at T1 (*R* = 0.4, *P* = 0.04).

The results were not influenced by the hemodynamic response to fluid loading. The increase in PaO_2_/FiO_2_ and LUS were not different between 13 patients whose CI increased by less than 10% and 19 patients whose CI increased by more than 10%.

## Discussion

The present study performed in patients with septic shock and ARDS shows that the hemodynamic benefit of fluid loading is associated with worsened lung aeration that does not match a decline in oxygenation. Following goal-directed fluid loading, CI and cardiac filling pressures immediately increased and rapidly returned to control values. It induced an immediate, slight but significant loss of lung aeration that worsened over time, whereas pulmonary shunt as well as regional pulmonary blood flow did not change. Arterial oxygenation increased at the end of fluid loading and returned to control values 40 minutes later.

Fluid loading-induced loss of lung aeration was detected by transthoracic ultrasound as an increase in LUS. In 87% of lung regions demonstrating ultrasound changes following fluid loading, new B lines appeared immediately or 40 minutes after volume expansion. B lines are vertical artifacts resulting from the abnormal interface existing between alveolar gas and excess of lung water (or tissue) and can be used for tracking aeration change [32]. Rapid appearance of multiple B lines (B1 lines) following fluid loading is strongly evocative of interstitial edema, whereas appearance of coalescent B lines (B2 lines) is suggestive of interstitial-alveolar edema. In the present study these changes were observed in less than 20% of examined lung areas, suggesting that fluid loading-induced interstitial-alveolar edema had a regional and heterogeneous distribution. Interestingly, the occurrence of regional lung edema was not associated with an increase in pulmonary shunt. As shown in Figure 6, despite the increase in CI, regional pulmonary blood flows within consolidated lung regions did not change, suggesting a predominant distribution in pulmonary vessels supplying normally aerated lung regions. As a matter of fact, between 40 and 50% of lung regions were normally aerated before fluid loading, leaving the possibility of a predominant distribution of the increase in CI in these regions.

Immediately after fluid loading, CI and arterial oxygenation significantly increased without change in pulmonary shunt. In fact, fluid loading-induced improvement in arterial oxygenation was related to a reduction in arteriovenous oxygen difference [33], whereas the most likely mechanism explaining fluid loading-induced deterioration of lung aeration was the slight but significant increase in PCWP in patients with injury of the alveolar capillary membrane. Many studies have demonstrated that even modest changes in PCWP can limit or promote the formation of interstitial-alveolar edema in experimental ARDS [32].

What are the clinical implications of our findings? When septic shock is associated with ARDS, the optimum fluid strategy remains uncertain and controversial: early vascular filling is critical for supporting hemodynamics and improving outcome in septic patients [12], whereas fluid loading may be deleterious for lung function [34-37]. A study performed in patients with acute lung injury secondary to septic shock, has proposed a biphasic fluid strategy [13]: fluid loading based on hemodynamic and oxygenation targets during the first 48 h and fluid restriction during the following days. The present study shows, however, that early fluid loading only transitorily improves hemodynamics and oxygenation, whereas it induces a progressive decrease in lung aeration detected by transthoracic LUS. This unexpected result confirms experimental data showing that LUS is much more sensitive than oxygenation changes to detect loss of lung aeration [38]. Therefore, by detection of aeration loss at the bedside, lung ultrasound could be a sensitive tool to assess the risk/benefit ratio of fluid loading in patients with septic shock and ARDS. Late consequences of fluid loading-induced worsening of lung aeration were not assessed in the present study. Three studies, however, have reported increased mortality associated with positive fluid balance at the early phase of ARDS. In the ARDSnet tidal volume study cohort, higher mortality was observed in patients with a positive fluid balance observed at day 1 and persisting at day 4 [36]. Higher mortality was also observed in patients with an increase in extravascular lung water indexed to predicted body weight at day 1 [39,40].

Several methodological limitations are to be discussed. First, the enrolled population was characterized by mild to moderate ARDS associated with septic shock caused mainly by ventilator-associated pneumonia observed predominantly in surgical patients. Extrapulmonary ARDS was present in less than 20% of patients. As previously demonstrated, ventilator-associated pneumonia is made of disseminated foci of pneumonia associated to large consolidations predominating in the lower lobes [6,41,42]. Therefore, the results of the present study may not apply to other causes of medical ARDS where high permeability-type alveolar edema is predominant or to more severe and extended forms of the disease. Second, measurements were time-limited and the late consequences of fluid loading-induced decrease in lung aeration were not assessed. The fact that lung aeration continued to decrease 40 minutes after the end of fluid loading, despite return to control values of hemodynamic parameters, indicates that fluid penetration within interstitial and alveolar spaces is a slow process. Third, the investigator who performed the three transthoracic ultrasound examinations was not aware of any other cardiorespiratory parameters but was not blind to the protocol timing. An overestimation of LUS could have then resulted. A majority of lung ultrasound examinations were recorded. *A posteriori* examination of videos by a second investigator unaware of protocol timing did not reveal any substantial bias. Fourth there are potential sources of error in counting B lines. Assessment of B lines depends on the type of probe, wavelength emission frequency and filter technique for artifact suppression. Therefore, LUS is only a semiquantitative evaluation of lung water and interstitial syndrome [32]. Last but not least, the high level of expertise required to detect small changes in LUS together with operator dependence may limit the clinical applicability of bedside transthoracic lung ultrasound for determining detrimental fluid loading.

## Conclusions

In summary, when septic shock is associated to ARDS, fluid loading performed within 48 h of onset transitorily improves hemodynamics and oxygenation. Ultrasound and hemodynamic data suggest that fluid loading produces regional interstitial-alveolar edema caused by an increase in pulmonary microvascular hydrostatic pressure. Due to the increase in cardiac index and the lack of increase in pulmonary shunt, arterial oxygenation increases, concealing from the clinician, the deterioration of lung aeration. As a consequence, transthoracic lung ultrasound may serve as a safeguard against excessive fluid loading.

## Key messages

•Transthoracic lung ultrasound is a non invasive, easily repeatable and useful diagnostic tool at the bedside in critically ill patients.

•At the early phase of ARDS associated with septic shock, bedside transthoracic lung ultrasound detects early worsening of lung aeration resulting from fluid loading.

•Simultaneously, hemodynamics and oxygenation improve and lung ultrasound may serve as a safeguard against excessive fluid loading.

## Abbreviations

A: peak velocity of late filling; ANOVA: analysis of variance; ARDS: acute respiratory distress syndrome; CI: cardiac index; CO: cardiac output; CVP: central venous pressure; D(A-v)O2: arteriovenous oxygen difference; DO2: oxygen delivery; DT: deceleration time of E; E: peak velocity of early filling; E/A: ratio of early (E) to and late (atrial) ventricular filling velocity; E/Ea: ratio of early transmitral flow velocity to early diastolic mitral annulus velocity; FiO2: fraction of inspired oxygen; Hb: hemoglobin; LUS: lung ultrasound score; PaCO2: arterial carbon dioxide tension; PaO2: arterial oxygen tension; PCWP: pulmonary capillary wedge pressure; PEEP: positive end-expiratory pressure; PVRI: pulmonary vascular resistance index; QVa/Qt: intrapulmonary shunt; SvO2: venous mixed oxygen saturation; SVRI: systemic vascular resistance index; T: time; VO2: oxygen consumption; VTIAo: aortic velocity time-integral.

## Competing interests

The authors have no competing interests.

## Authors’ contributions

FPC was directly involved in the conception of the protocol, acquired all data, analyzed and drafted the manuscript. AM, CA, HB, and GJZ participated to the design of the study, supervised the acquisition of ultrasound and echocardiographic data and participated to the writing of the manuscript. QL and WJG performed the statistical analysis and participated in the draft of the manuscript. JOCAJ made the study possible, was involved in the conception of the protocol and participated in the writing of the manuscript and JJR designed the research protocol, made a determinant contribution to analysis and interpretation of data, made a critical contribution to the writing of the manuscript and takes responsibility for the integrity of the data and the accuracy of the data analysis. All authors read and approved the final manuscript.
